# Crystal structure and Hirshfeld surface analysis of (3*S*,3a*R*,6a*S*)-3-(1,3-diphenyl-1*H*-pyrazol-4-yl)-5-(4-meth­oxy­phen­yl)-2-phenyl-3,3a,4,5,6,6a-hexa­hydro-2*H*-pyrrolo­[3,4-*d*][1,2]oxazole-4,6-dione

**DOI:** 10.1107/S2056989021002358

**Published:** 2021-03-05

**Authors:** Shaaban K. Mohamed, Awad I. Said, Joel T. Mague, Talaat I. El-Emary, Mehmet Akkurt, Sahar M. I. Elgarhy

**Affiliations:** a Chemistry and Environmental Division, Manchester Metropolitan University, Manchester, M1 5GD, England; b Chemistry Department, Faculty of Science, Minia University, 61519 El-Minia, Egypt; c Chemistry Department, Faculty of Science, Assuit University, Egypt; d Department of Chemistry, Tulane University, New Orleans, LA 70118, USA; e Department of Physics, Faculty of Sciences, Erciyes University, 38039 Kayseri, Turkey; f Faculty of Science, Department of Bio Chemistry, Beni Suef University, Beni Suef, Egypt

**Keywords:** crystal structure, pyrrole, oxazole, hydrogen bond, C—H⋯π(ring), Hirshfeld surface analysis

## Abstract

In the title compound, C_33_H_26_N_4_O_4_, the two fused five-membered rings and their N-bound aromatic substituents form a pincer-like motif. The relative conformations about the three chiral carbon atoms are established. In the crystal, a combination of C—H⋯O and C—H⋯N hydrogen bonds and C—H⋯π(ring) inter­actions leads to the formation of layers parallel to the *bc* plane. A Hirshfeld surface analysis indicates that the most significant contributions to the crystal packing are from H⋯H (44.3%), C⋯H/H⋯C (29.8%) and O⋯H/H⋯O (15.0%) contacts.

## Abstract 2

The relative conformations about the three chiral carbon atoms are established. The two fused five-membered rings and their N-bound aromatic substituents form a pincer-like motif. In the crystal, a combination of C—H⋯O and C—H⋯N hydrogen bonds and C—H⋯π(ring) inter­actions leads to the formation of layers parallel to the *bc* plane with the di­phenyl­pyrrole groups protruding from both sides of the layers.

## Chemical context   

Oxazole scaffold compounds currently find application in medicinal drugs such as Aleglitazar (anti­diabetic), Ditazole (platelets aggregation inhibitor), Mubritinib (tyrosine kinase inhibitor), and Oxaprozin (COX-2 inhibitor) (Kakkar *et al.*, 2018 ▸). In addition they show anti-microbial (Tomi *et al.*, 2015 ▸) and anti-cancer (Liu *et al.*, 2009 ▸) activity. In this context, we determined the crystal structure of the title compound.
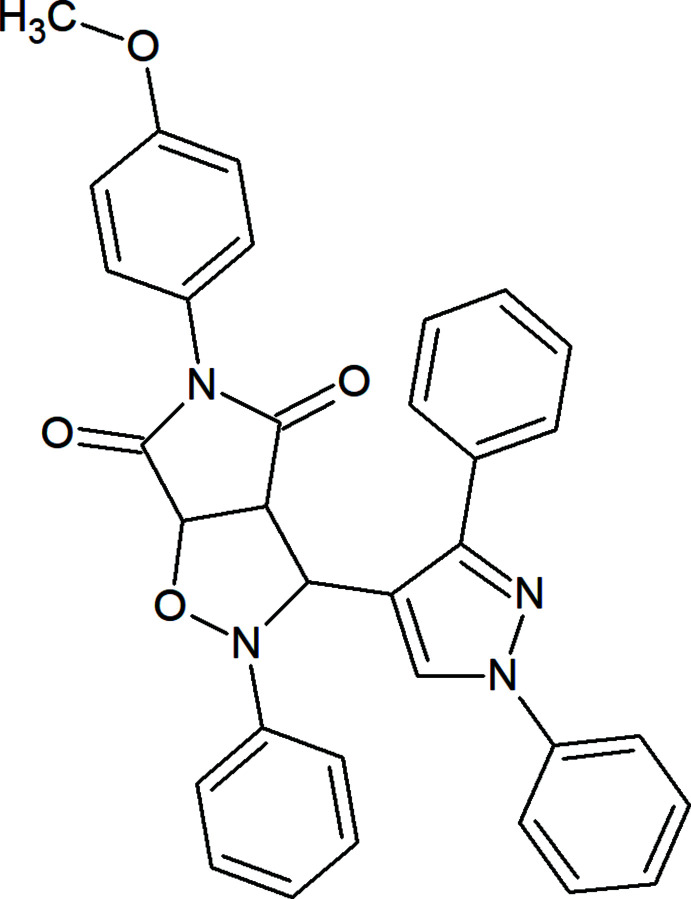


## Structural commentary   

**Figure 1:**
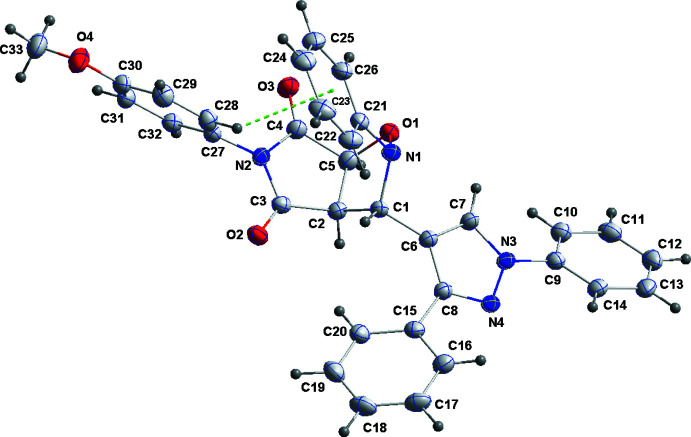
The title mol­ecule with labeling scheme and 50% probability ellipsoids. The intra­molecular C—H⋯π(ring) inter­action is shown by a dashed line.

A puckering analysis of the oxazole fragment (Cremer & Pople, 1975 ▸) of the title mol­ecule (Fig. 1 ▸) indicates it to have an envelope conformation on N1 with *Q*(2) = 0.3541 (14) Å and φ(2) = 223.2 (2)°. The pyrrolo­oxazole fragment is folded along the C2⋯C5 axis by 62.63 (8)° while the dihedral angle between the C2/C3/N2/C4/C5 and C27–C32 rings is 67.11 (8)°. The C9–C14 and C15–C20 rings are inclined to the C6/C7/N3/N4/C8 ring by 32.32 (9) and 33.52 (9)°, respectively.

## Supra­molecular features   

**Figure 2:**
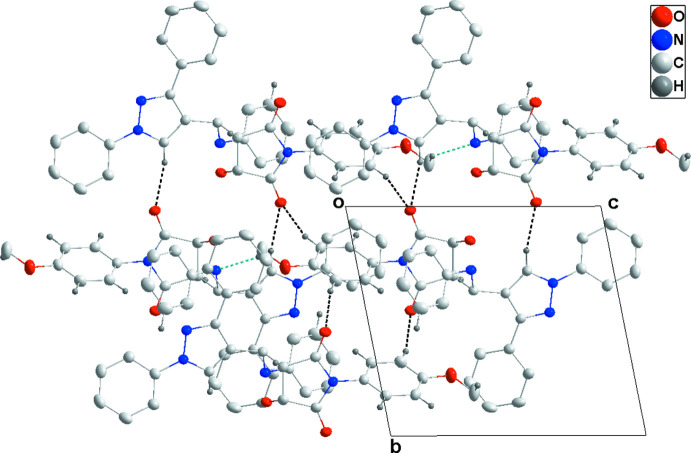
Detail of the inter­molecular C—H⋯O and C—H⋯N hydrogen bonds (black and light-blue dashed lines, respectively) viewed along the *a*-axis direction.

**Figure 4:**
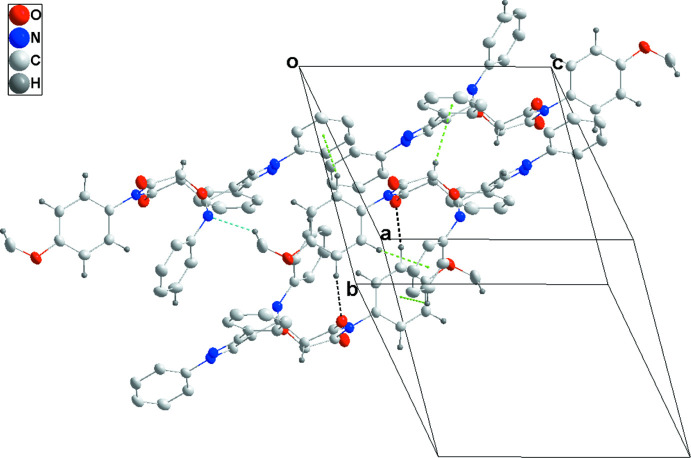
Detail of the C—H⋯π(ring) inter­actions (green dashed lines). C—H⋯O and C—H⋯N hydrogen bonds between the involved mol­ecules are depicted as in Fig. 2 ▸.

In the crystal, the mol­ecules form chains along the *c*-axis direction through C33—H33*A*⋯N1 hydrogen bonds. On one side, the chains are connected by C7—H7⋯O3 and C31—H31⋯O3 hydrogen bonds and on the other by inversion-related C29—H29⋯O2 hydrogen bonds, forming layers parallel to the *bc* plane (Table 1 ▸ and Figs. 2 ▸ and 3 ▸). The layer formation is bolstered by C2—H2⋯*Cg*5, C23—H23⋯*Cg*7, C28—H28⋯*Cg*6 and C32—H32⋯*Cg*4 inter­actions (Table 1 ▸ and Fig. 4 ▸). The di­phenyl­pyrrole groups protrude from both faces of the layers.

## Hirshfeld surface analysis   

**Figure 5:**
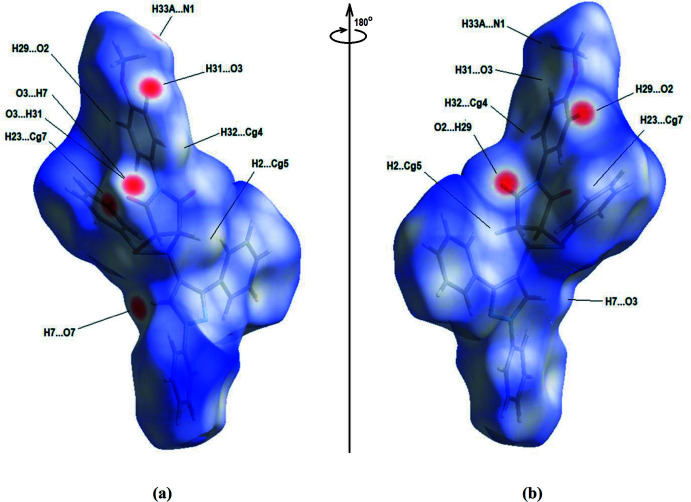
(*a*) Front and (*b*) back sides of the three-dimensional Hirshfeld surface of the title compound plotted over *d*_norm_ in the range −0.3067 to 1.6634 a.u.

A Hirshfeld surface analysis and the associated two-dimensional fingerprint plots were performed with *CrystalExplorer17* (Turner *et al.*, 2017 ▸) for the identification of the inter­molecular inter­actions in the title compound. Fig. 5 ▸(*a*) and Fig. 5 ▸(*b*) show the front and back sides of the three-dimensional Hirshfeld surface of the title compound plotted over *d*_norm_ in the range −0.3067 to 1.6634 a.u. The red spots highlight the inter­atomic contacts, including the C—H⋯O hydrogen bonds.

**Figure 6:**
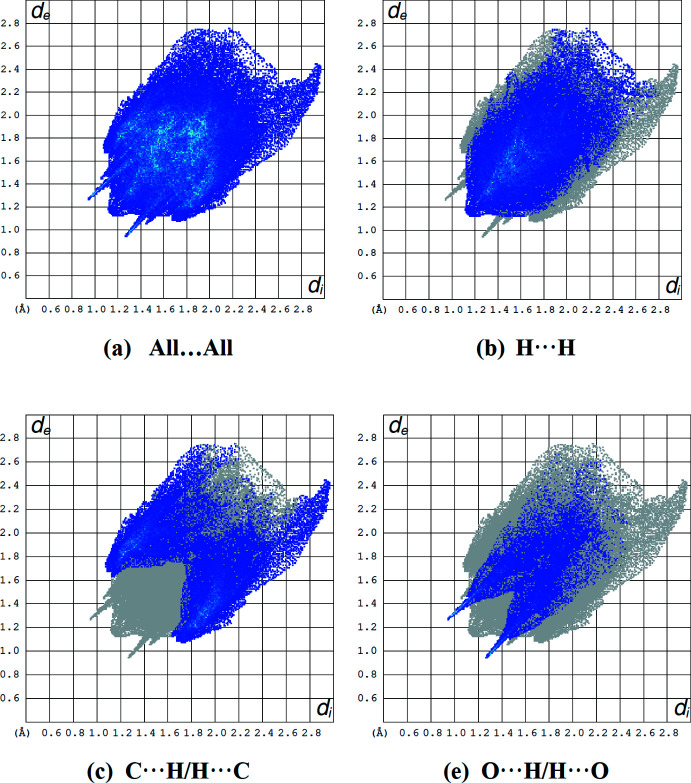
A view of the two-dimensional fingerprint plots for the title compound, showing (*a*) all inter­actions, and delineated into (*b*) H⋯H, (*c*) C⋯H/H⋯C, and (*d*) O⋯H/H⋯O inter­actions. The *d*_i_ and *d*_e_ values are the closest inter­nal and external distances (in Å) from given points on the Hirshfeld surface.

The overall two-dimensional fingerprint plot, and those delineated into H⋯H (44.3%), C⋯H/H⋯C (29.8%) and O⋯H/H⋯O (15.0%) contacts (Table 2 ▸) are illustrated in Fig. 6 ▸ *a*–*d*, respectively. The other minor contributions to the Hirshfeld surface are by N⋯H/H⋯N (6.5%), C⋯C (1.8%), O⋯C/C⋯O (1.3%), N⋯O/O⋯N (0.5%), O⋯O (0.5%) and N⋯C/C⋯N (0.3%) contacts. The large number of H⋯H, C⋯H/H⋯C and O⋯H/H⋯O inter­actions suggest that van der Waals inter­actions and hydrogen bonding play the major roles in the crystal packing (Hathwar *et al.*, 2015 ▸).

## Database survey   

In the crystal structure of the related compound (6*R*,7a*S*)-1,3,5,6,7,7a-hexa­hydro-3-(2-hy­droxy­phen­yl)-1,1-di­phenyl­pyr­rolo­(1,2-*c*)(1,3)oxazol-6-ol [CSD Groom *et al.*, 2016 ▸) refcode FOMYEM: Shen *et al.*, 2005 ▸], the mol­ecules are connected by O—H⋯O and C—H⋯O hydrogen bonds, forming chains along [010]. The chains further inter­act through C—H⋯O hydrogen bonds, stacking along [100]. In the third direction [001], there are only weak van der Waals inter­actions, which explains the thin plate habit of the crystals.

## Synthesis and crystallization   

A mixture of *N*-(4-meth­oxy­phen­yl) male­imide (0.6 g, 3 mmol) and (*Z*)-*N*-[(1,3-diphenyl-1*H*-pyrazol-4-yl)methyl­ene]benz­en­amine oxide (1.1 g, 3 mmol) in toluene (15 ml) was heated at 373 K under reflux for 24 h, the reaction was monitored by TLC. The endo isomer was filtered off as a major product. The title compound was recrystallized from a mixture of toluene and petroleum ether as colorless crystals in 60% yield; mp: 469–471 K.

## Refinement   

Crystal data, data collection and structure refinement details are summarized in Table 3 ▸. H atoms on C atoms were located in a difference-Fourier map and were freely refined.

## Supplementary Material

Crystal structure: contains datablock(s) global, I. DOI: 10.1107/S2056989021002358/ex2041sup1.cif

Structure factors: contains datablock(s) I. DOI: 10.1107/S2056989021002358/ex2041Isup2.hkl

Supporting information file. DOI: 10.1107/S2056989021002358/ex2041Isup3.cml

CCDC reference: 2067379

Additional supporting information:  crystallographic information; 3D view; checkCIF report

## Figures and Tables

**Figure 3:**
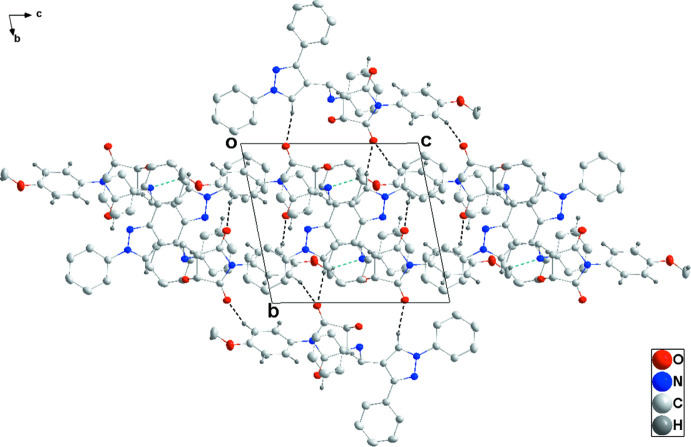
Packing viewed along the *a*-axis direction with C—H⋯O and C—H⋯N hydrogen bonds depicted as in Fig. 2 ▸. The C—H⋯π(ring) inter­actions are omitted for clarity.

**Table 1:** Hydrogen-bond geometry (Å, °) *Cg*4, *Cg*5, *Cg*6 and *Cg*7 are the centroids of the C9–C14, C15–C20, C21–C26 and C27–C32 benzene rings, respectively.

*D*—H⋯*A*	*D*—H	H⋯*A*	*D*⋯*A*	*D*—H⋯*A*
C2—H2⋯*Cg*5^i^	0.992 (16)	2.817 (19)	3.7292 (17)	153.2 (12)
C7—H7⋯O3^ii^	0.954 (15)	2.329 (15)	3.2666 (16)	167.4 (12)
C22—H22⋯O4^iii^	0.981 (17)	2.648 (17)	3.4762 (18)	142.3 (13)
C23—H23⋯*Cg*7^iii^	0.970 (19)	2.97 (2)	3.6086 (18)	124.2 (16)
C28—H28⋯*Cg*6	0.986 (18)	2.66 (2)	3.4522 (17)	138.1 (14)
C29—H29⋯O2^iii^	0.950 (18)	2.374 (18)	3.2803 (17)	159.3 (14)
C31—H31⋯O3^iv^	0.985 (17)	2.360 (17)	3.3133 (17)	162.5 (14)
C32—H32⋯*Cg*4^v^	0.979 (16)	2.71 (2)	3.3750 (18)	125.3 (12)
C33—H33*A*⋯N1^v^	0.98 (2)	2.58 (2)	3.393 (2)	140.6 (17)

Symmetry codes: (i) -x, -y+1, -z+1; (ii) -x+1, -y, -z+1; (iii) -x+1, -y+1, -z; (iv) -x+1, -y, -z; (v) x, y, z-1.

**Table 2:** Summary of short inter­atomic contacts (Å) in the title compound

Contact	Distance	Symmetry operation
H7⋯O3	2.33	1 − *x*, −*y*, 1 − *z*
H29⋯O2	2.37	1 − *x*, 1 − *y*, −*z*
H31⋯O3	2.36	1 − *x*, −*y*, −*z*
N1⋯H33*A*	2.58	*x*, *y*, 1 + *z*
H16⋯H13	2.41	−*x*, 1 − *y*, 2 − *z*
H2⋯C16	2.84	−*x*, 1 − *y*, 1 − *z*
C13⋯H24	2.82	−1 + *x*, *y*, 1 + *z*
H17⋯H17	2.56	−*x*, 2 − *y*, 1 − *z*
H24⋯H14	2.57	1 − *x*, 1 − *y*, 1 − *z*

**Table 3:** Experimental details

Crystal data	
Chemical formula	C_33_H_26_N_4_O_4_
*M*_r_	542.58
Crystal system, space group	Triclinic, *P*\overline{1}
Temperature (K)	150
*a*, *b*, *c* (Å)	11.5014 (3), 11.5340 (3), 11.7878 (3)
α, β, γ (°)	73.567 (1), 74.613 (1), 64.218 (1)
*V* (Å^3^)	1332.10 (6)
*Z*	2
Radiation type	Cu *K*α
μ (mm^−1^)	0.73
Crystal size (mm)	0.13 × 0.11 × 0.05

Data collection	
Diffractometer	Bruker D8 VENTURE PHOTON 100 CMOS
Absorption correction	Multi-scan (*SADABS*; Bruker, 2016 ▸)
*T*_min_, *T*_max_	0.86, 0.96
No. of measured, independent and observed [*I* > 2σ(*I*)] reflections	10211, 4940, 4357
*R*_int_	0.027
(sin θ/λ)_max_ (Å^−1^)	0.617

Refinement	
*R*[*F* ^2^ > 2σ(*F* ^2^)], *wR*(*F* ^2^), *S*	0.037, 0.098, 1.05
No. of reflections	4940
No. of parameters	475
H-atom treatment	All H-atom parameters refined
Δρ_max_, Δρ_min_ (e Å^−3^)	0.23, −0.20

Computer programs: *APEX3* and *SAINT* (Bruker, 2016 ▸), *SAINT* (Bruker, 2016 ▸), *SHELXT* (Sheldrick, 2015*a* ▸), *SHELXL2016/6* (Sheldrick, 2015*b* ▸), *DIAMOND* (Brandenburg & Putz, 2012 ▸), and *SHELXTL* (Sheldrick, 2008 ▸).

## supplementary crystallographic information

### Crystal data

**Table:**

C_33_H_26_N_4_O_4_	*Z* = 2
*M**_r_* = 542.58	*F*(000) = 568
Triclinic, *P*1	*D*_x_ = 1.353 Mg m^−^^3^
*a* = 11.5014 (3) Å	Cu *K*α radiation, λ = 1.54178 Å
*b* = 11.5340 (3) Å	Cell parameters from 8367 reflections
*c* = 11.7878 (3) Å	θ = 4.0–72.0°
α = 73.567 (1)°	µ = 0.73 mm^−^^1^
β = 74.613 (1)°	*T* = 150 K
γ = 64.218 (1)°	Block, colourless
*V* = 1332.10 (6) Å^3^	0.13 × 0.11 × 0.05 mm

### Data collection

**Table:**

Bruker D8 VENTURE PHOTON 100 CMOS diffractometer	4940 independent reflections
Radiation source: INCOATEC IµS micro–focus source	4357 reflections with *I* > 2σ(*I*)
Mirror monochromator	*R*_int_ = 0.027
Detector resolution: 10.4167 pixels mm^-1^	θ_max_ = 72.0°, θ_min_ = 4.0°
ω scans	*h* = −14→14
Absorption correction: multi-scan (*SADABS*; Bruker, 2016)	*k* = −14→13
*T*_min_ = 0.86, *T*_max_ = 0.96	*l* = −13→14
10211 measured reflections	

### Refinement

**Table:**

Refinement on *F*^2^	Secondary atom site location: difference Fourier map
Least-squares matrix: full	Hydrogen site location: difference Fourier map
*R*[*F*^2^ > 2σ(*F*^2^)] = 0.037	All H-atom parameters refined
*wR*(*F*^2^) = 0.098	*w* = 1/[σ^2^(*F*_o_^2^) + (0.0455*P*)^2^ + 0.394*P*] where *P* = (*F*_o_^2^ + 2*F*_c_^2^)/3
*S* = 1.05	(Δ/σ)_max_ < 0.001
4940 reflections	Δρ_max_ = 0.23 e Å^−^^3^
475 parameters	Δρ_min_ = −0.20 e Å^−^^3^
0 restraints	Extinction correction: *SHELXL* 2016/6 (Sheldrick, 2015*b*), Fc^*^=kFc[1+0.001xFc^2^λ^3^/sin(2θ)]^-1/4^
Primary atom site location: structure-invariant direct methods	Extinction coefficient: 0.0058 (5)

### Special details

**Table:**

Geometry. All esds (except the esd in the dihedral angle between two l.s. planes) are estimated using the full covariance matrix. The cell esds are taken into account individually in the estimation of esds in distances, angles and torsion angles; correlations between esds in cell parameters are only used when they are defined by crystal symmetry. An approximate (isotropic) treatment of cell esds is used for estimating esds involving l.s. planes.
Refinement. Refinement of F^2^ against ALL reflections. The weighted R-factor wR and goodness of fit S are based on F^2^, conventional R-factors R are based on F, with F set to zero for negative F^2^. The threshold expression of F^2^ > 2sigma(F^2^) is used only for calculating R-factors(gt) etc. and is not relevant to the choice of reflections for refinement. R-factors based on F^2^ are statistically about twice as large as those based on F, and R- factors based on ALL data will be even larger.

### Fractional atomic coordinates and isotropic or equivalent isotropic displacement parameters (Å^2^)

**Table:**

	*x*	*y*	*z*	*U*_iso_*/*U*_eq_	
O1	0.46877 (9)	0.14792 (8)	0.46118 (8)	0.0270 (2)	
O2	0.26199 (10)	0.45329 (9)	0.17596 (9)	0.0302 (2)	
O3	0.52260 (10)	0.01734 (9)	0.25051 (9)	0.0336 (2)	
O4	0.67552 (11)	0.26355 (11)	−0.28302 (9)	0.0390 (3)	
N1	0.46935 (11)	0.27693 (10)	0.44250 (10)	0.0245 (2)	
N2	0.40487 (11)	0.23670 (10)	0.18438 (10)	0.0236 (2)	
N3	0.18502 (11)	0.33463 (10)	0.75289 (10)	0.0246 (2)	
N4	0.10816 (10)	0.46224 (10)	0.71745 (10)	0.0243 (2)	
C1	0.33181 (12)	0.36685 (12)	0.43619 (11)	0.0228 (3)	
H1	0.3304 (15)	0.4527 (15)	0.3909 (13)	0.024 (4)*	
C2	0.28461 (13)	0.30151 (12)	0.36855 (12)	0.0245 (3)	
H2	0.1901 (16)	0.3188 (15)	0.3945 (13)	0.026 (4)*	
C3	0.31112 (12)	0.34491 (12)	0.23320 (12)	0.0238 (3)	
C4	0.44410 (13)	0.12338 (12)	0.27068 (12)	0.0258 (3)	
C5	0.37528 (13)	0.15754 (13)	0.39352 (12)	0.0265 (3)	
H5	0.3347 (16)	0.0972 (15)	0.4409 (14)	0.028 (4)*	
C6	0.25513 (12)	0.37807 (12)	0.55967 (12)	0.0235 (3)	
C7	0.27413 (13)	0.28120 (13)	0.66093 (12)	0.0256 (3)	
H7	0.3367 (15)	0.1930 (15)	0.6739 (13)	0.022 (4)*	
C8	0.14996 (12)	0.48910 (12)	0.59946 (11)	0.0228 (3)	
C9	0.16696 (12)	0.27185 (13)	0.87539 (12)	0.0253 (3)	
C10	0.19210 (14)	0.13870 (14)	0.90401 (13)	0.0289 (3)	
H10	0.2233 (16)	0.0892 (15)	0.8422 (14)	0.028 (4)*	
C11	0.17540 (14)	0.07874 (15)	1.02412 (14)	0.0345 (3)	
H11	0.1937 (18)	−0.0118 (19)	1.0384 (16)	0.044 (5)*	
C12	0.13366 (14)	0.15145 (16)	1.11359 (13)	0.0355 (3)	
H12	0.1232 (18)	0.1059 (18)	1.1967 (17)	0.044 (5)*	
C13	0.10923 (14)	0.28455 (15)	1.08380 (13)	0.0331 (3)	
H13	0.0795 (18)	0.3375 (17)	1.1455 (16)	0.043 (5)*	
C14	0.12613 (13)	0.34511 (14)	0.96421 (12)	0.0281 (3)	
H14	0.1116 (16)	0.4389 (17)	0.9412 (14)	0.034 (4)*	
C15	0.08571 (12)	0.62188 (12)	0.53116 (12)	0.0241 (3)	
C16	0.03690 (14)	0.72978 (13)	0.58761 (13)	0.0285 (3)	
H16	0.0510 (16)	0.7157 (15)	0.6670 (15)	0.028 (4)*	
C17	−0.02833 (15)	0.85474 (14)	0.52723 (15)	0.0351 (3)	
H17	−0.0626 (18)	0.9299 (18)	0.5673 (15)	0.041 (5)*	
C18	−0.04373 (15)	0.87516 (15)	0.40921 (15)	0.0386 (4)	
H18	−0.088 (2)	0.962 (2)	0.3644 (17)	0.052 (5)*	
C19	0.00554 (15)	0.76944 (15)	0.35259 (14)	0.0361 (3)	
H19	−0.0036 (18)	0.7840 (17)	0.2689 (17)	0.043 (5)*	
C20	0.06899 (13)	0.64321 (14)	0.41334 (13)	0.0289 (3)	
H20	0.1000 (16)	0.5690 (16)	0.3741 (15)	0.032 (4)*	
C21	0.56878 (12)	0.29685 (13)	0.34556 (12)	0.0250 (3)	
C22	0.56937 (14)	0.42275 (14)	0.30470 (14)	0.0318 (3)	
H22	0.5019 (17)	0.4971 (17)	0.3399 (15)	0.033 (4)*	
C23	0.66806 (15)	0.44335 (16)	0.21469 (15)	0.0373 (3)	
H23	0.6673 (18)	0.5311 (19)	0.1846 (16)	0.043 (5)*	
C24	0.76798 (14)	0.33984 (16)	0.16605 (15)	0.0375 (3)	
H24	0.838 (2)	0.3552 (18)	0.1016 (17)	0.049 (5)*	
C25	0.76971 (14)	0.21467 (15)	0.20983 (14)	0.0347 (3)	
H25	0.8426 (18)	0.1395 (18)	0.1773 (16)	0.040 (5)*	
C26	0.67152 (13)	0.19190 (14)	0.29954 (13)	0.0295 (3)	
H26	0.6735 (17)	0.1023 (17)	0.3297 (15)	0.035 (4)*	
C27	0.46908 (12)	0.24495 (12)	0.06130 (11)	0.0233 (3)	
C28	0.54580 (14)	0.31853 (13)	0.01970 (12)	0.0279 (3)	
H28	0.5515 (17)	0.3678 (17)	0.0727 (16)	0.038 (4)*	
C29	0.61394 (14)	0.32202 (14)	−0.09549 (13)	0.0310 (3)	
H29	0.6701 (17)	0.3686 (17)	−0.1243 (15)	0.037 (4)*	
C30	0.60485 (13)	0.25230 (13)	−0.16986 (12)	0.0281 (3)	
C31	0.52736 (13)	0.17974 (13)	−0.12874 (12)	0.0272 (3)	
H31	0.5212 (16)	0.1302 (16)	−0.1811 (15)	0.033 (4)*	
C32	0.45934 (13)	0.17575 (13)	−0.01174 (12)	0.0251 (3)	
H32	0.4043 (16)	0.1249 (15)	0.0197 (14)	0.026 (4)*	
C33	0.67512 (19)	0.1895 (2)	−0.36073 (16)	0.0462 (4)	
H33A	0.587 (2)	0.219 (2)	−0.3788 (19)	0.063 (6)*	
H33B	0.710 (2)	0.090 (2)	−0.3253 (19)	0.062 (6)*	
H33C	0.731 (2)	0.209 (2)	−0.4359 (19)	0.056 (6)*	

### Atomic displacement parameters (Å^2^)

**Table:**

	*U*^11^	*U*^22^	*U*^33^	*U*^12^	*U*^13^	*U*^23^
O1	0.0298 (5)	0.0207 (4)	0.0256 (5)	−0.0064 (4)	−0.0054 (4)	−0.0018 (4)
O2	0.0324 (5)	0.0225 (5)	0.0320 (5)	−0.0048 (4)	−0.0113 (4)	−0.0039 (4)
O3	0.0408 (6)	0.0213 (5)	0.0303 (5)	−0.0045 (4)	−0.0039 (4)	−0.0070 (4)
O4	0.0393 (6)	0.0507 (7)	0.0265 (5)	−0.0213 (5)	0.0036 (5)	−0.0087 (5)
N1	0.0233 (5)	0.0207 (5)	0.0256 (6)	−0.0052 (4)	−0.0043 (4)	−0.0041 (4)
N2	0.0254 (5)	0.0220 (5)	0.0219 (6)	−0.0075 (4)	−0.0032 (4)	−0.0056 (4)
N3	0.0244 (5)	0.0212 (5)	0.0224 (6)	−0.0050 (4)	−0.0017 (4)	−0.0040 (4)
N4	0.0231 (5)	0.0218 (5)	0.0244 (6)	−0.0057 (4)	−0.0032 (4)	−0.0048 (4)
C1	0.0214 (6)	0.0211 (6)	0.0221 (6)	−0.0048 (5)	−0.0033 (5)	−0.0044 (5)
C2	0.0218 (6)	0.0245 (7)	0.0250 (7)	−0.0069 (5)	−0.0019 (5)	−0.0067 (5)
C3	0.0220 (6)	0.0224 (6)	0.0276 (7)	−0.0071 (5)	−0.0063 (5)	−0.0062 (5)
C4	0.0286 (7)	0.0221 (6)	0.0263 (7)	−0.0100 (5)	−0.0041 (5)	−0.0044 (5)
C5	0.0291 (7)	0.0232 (6)	0.0256 (7)	−0.0105 (6)	−0.0019 (6)	−0.0041 (5)
C6	0.0227 (6)	0.0225 (6)	0.0232 (6)	−0.0066 (5)	−0.0023 (5)	−0.0062 (5)
C7	0.0255 (6)	0.0224 (7)	0.0234 (7)	−0.0049 (5)	−0.0010 (5)	−0.0061 (5)
C8	0.0221 (6)	0.0220 (6)	0.0237 (6)	−0.0075 (5)	−0.0031 (5)	−0.0062 (5)
C9	0.0208 (6)	0.0268 (7)	0.0240 (7)	−0.0074 (5)	−0.0026 (5)	−0.0024 (5)
C10	0.0273 (7)	0.0271 (7)	0.0283 (7)	−0.0083 (5)	−0.0041 (6)	−0.0038 (6)
C11	0.0282 (7)	0.0302 (8)	0.0356 (8)	−0.0083 (6)	−0.0051 (6)	0.0024 (6)
C12	0.0273 (7)	0.0434 (9)	0.0257 (7)	−0.0110 (6)	−0.0023 (6)	0.0019 (6)
C13	0.0236 (6)	0.0436 (8)	0.0263 (7)	−0.0091 (6)	0.0000 (6)	−0.0088 (6)
C14	0.0230 (6)	0.0298 (7)	0.0275 (7)	−0.0077 (5)	−0.0003 (5)	−0.0072 (5)
C15	0.0191 (6)	0.0227 (6)	0.0276 (7)	−0.0064 (5)	−0.0023 (5)	−0.0051 (5)
C16	0.0284 (7)	0.0255 (7)	0.0289 (7)	−0.0103 (6)	0.0002 (6)	−0.0060 (5)
C17	0.0313 (7)	0.0218 (7)	0.0439 (9)	−0.0061 (6)	−0.0002 (6)	−0.0066 (6)
C18	0.0310 (7)	0.0262 (7)	0.0467 (9)	−0.0049 (6)	−0.0104 (7)	0.0040 (6)
C19	0.0314 (7)	0.0390 (8)	0.0339 (8)	−0.0103 (6)	−0.0133 (6)	0.0008 (6)
C20	0.0240 (6)	0.0301 (7)	0.0314 (7)	−0.0074 (5)	−0.0071 (6)	−0.0068 (6)
C21	0.0212 (6)	0.0284 (7)	0.0240 (6)	−0.0072 (5)	−0.0069 (5)	−0.0039 (5)
C22	0.0249 (7)	0.0302 (7)	0.0404 (8)	−0.0098 (6)	−0.0064 (6)	−0.0075 (6)
C23	0.0283 (7)	0.0354 (8)	0.0483 (9)	−0.0157 (6)	−0.0081 (7)	−0.0013 (7)
C24	0.0238 (7)	0.0463 (9)	0.0398 (9)	−0.0157 (6)	−0.0042 (6)	−0.0021 (7)
C25	0.0219 (7)	0.0401 (8)	0.0354 (8)	−0.0068 (6)	−0.0023 (6)	−0.0084 (6)
C26	0.0226 (6)	0.0290 (7)	0.0310 (7)	−0.0048 (5)	−0.0057 (6)	−0.0044 (6)
C27	0.0231 (6)	0.0228 (6)	0.0209 (6)	−0.0060 (5)	−0.0039 (5)	−0.0043 (5)
C28	0.0322 (7)	0.0268 (7)	0.0274 (7)	−0.0127 (6)	−0.0078 (6)	−0.0045 (5)
C29	0.0332 (7)	0.0310 (7)	0.0312 (7)	−0.0173 (6)	−0.0061 (6)	−0.0007 (6)
C30	0.0259 (6)	0.0300 (7)	0.0224 (7)	−0.0077 (5)	−0.0028 (5)	−0.0026 (5)
C31	0.0274 (7)	0.0284 (7)	0.0258 (7)	−0.0083 (5)	−0.0053 (6)	−0.0086 (5)
C32	0.0236 (6)	0.0236 (6)	0.0280 (7)	−0.0084 (5)	−0.0047 (5)	−0.0055 (5)
C33	0.0416 (9)	0.0681 (13)	0.0281 (8)	−0.0206 (9)	0.0018 (7)	−0.0166 (8)

### Geometric parameters (Å, º)

**Table:**

O1—N1	1.4440 (14)	C14—H14	0.986 (17)
O1—C5	1.4511 (16)	C15—C20	1.3905 (19)
O2—C3	1.2032 (16)	C15—C16	1.4034 (18)
O3—C4	1.2073 (16)	C16—C17	1.382 (2)
O4—C30	1.3694 (17)	C16—H16	0.949 (16)
O4—C33	1.420 (2)	C17—C18	1.390 (2)
N1—C21	1.4329 (17)	C17—H17	0.982 (18)
N1—C1	1.4748 (16)	C18—C19	1.383 (2)
N2—C4	1.3875 (17)	C18—H18	0.97 (2)
N2—C3	1.3993 (16)	C19—C20	1.389 (2)
N2—C27	1.4400 (17)	C19—H19	0.978 (19)
N3—N4	1.3559 (15)	C20—H20	0.972 (17)
N3—C7	1.3580 (17)	C21—C26	1.3939 (19)
N3—C9	1.4257 (16)	C21—C22	1.397 (2)
N4—C8	1.3389 (17)	C22—C23	1.387 (2)
C1—C6	1.4965 (18)	C22—H22	0.981 (17)
C1—C2	1.5570 (17)	C23—C24	1.385 (2)
C1—H1	0.978 (16)	C23—H23	0.970 (19)
C2—C5	1.5204 (18)	C24—C25	1.382 (2)
C2—C3	1.5205 (18)	C24—H24	0.99 (2)
C2—H2	0.992 (16)	C25—C26	1.389 (2)
C4—C5	1.5237 (19)	C25—H25	0.994 (18)
C5—H5	0.969 (16)	C26—H26	0.985 (17)
C6—C7	1.3757 (19)	C27—C32	1.3815 (18)
C6—C8	1.4192 (17)	C27—C28	1.3877 (19)
C7—H7	0.954 (15)	C28—C29	1.375 (2)
C8—C15	1.4742 (18)	C28—H28	0.986 (18)
C9—C14	1.3874 (19)	C29—C30	1.395 (2)
C9—C10	1.3886 (19)	C29—H29	0.950 (18)
C10—C11	1.392 (2)	C30—C31	1.386 (2)
C10—H10	0.951 (16)	C31—C32	1.393 (2)
C11—C12	1.388 (2)	C31—H31	0.985 (17)
C11—H11	0.945 (19)	C32—H32	0.979 (16)
C12—C13	1.390 (2)	C33—H33A	0.98 (2)
C12—H12	0.976 (19)	C33—H33B	1.03 (2)
C13—C14	1.389 (2)	C33—H33C	0.98 (2)
C13—H13	0.978 (18)		
			
N1—O1—C5	107.88 (9)	C13—C14—H14	121.2 (9)
C30—O4—C33	117.49 (12)	C20—C15—C16	118.93 (13)
C21—N1—O1	112.08 (10)	C20—C15—C8	121.98 (12)
C21—N1—C1	118.58 (10)	C16—C15—C8	119.08 (12)
O1—N1—C1	104.26 (9)	C17—C16—C15	120.27 (14)
C4—N2—C3	112.73 (11)	C17—C16—H16	120.8 (9)
C4—N2—C27	122.68 (11)	C15—C16—H16	119.0 (9)
C3—N2—C27	123.94 (10)	C16—C17—C18	120.35 (14)
N4—N3—C7	112.51 (11)	C16—C17—H17	119.9 (10)
N4—N3—C9	120.07 (10)	C18—C17—H17	119.7 (10)
C7—N3—C9	127.42 (11)	C19—C18—C17	119.65 (14)
C8—N4—N3	104.61 (10)	C19—C18—H18	118.2 (12)
N1—C1—C6	110.28 (10)	C17—C18—H18	122.2 (12)
N1—C1—C2	103.54 (10)	C18—C19—C20	120.39 (14)
C6—C1—C2	112.28 (11)	C18—C19—H19	119.6 (11)
N1—C1—H1	108.2 (9)	C20—C19—H19	120.0 (11)
C6—C1—H1	110.3 (9)	C19—C20—C15	120.39 (13)
C2—C1—H1	112.0 (9)	C19—C20—H20	119.9 (10)
C5—C2—C3	105.11 (10)	C15—C20—H20	119.7 (10)
C5—C2—C1	103.42 (10)	C26—C21—C22	119.39 (13)
C3—C2—C1	112.44 (10)	C26—C21—N1	121.46 (12)
C5—C2—H2	114.6 (9)	C22—C21—N1	118.85 (12)
C3—C2—H2	109.0 (9)	C23—C22—C21	119.91 (14)
C1—C2—H2	112.1 (9)	C23—C22—H22	119.0 (10)
O2—C3—N2	124.77 (12)	C21—C22—H22	121.0 (10)
O2—C3—C2	126.87 (12)	C24—C23—C22	120.74 (14)
N2—C3—C2	108.36 (10)	C24—C23—H23	119.2 (11)
O3—C4—N2	125.05 (13)	C22—C23—H23	120.0 (11)
O3—C4—C5	126.56 (12)	C25—C24—C23	119.17 (14)
N2—C4—C5	108.36 (11)	C25—C24—H24	120.5 (11)
O1—C5—C2	106.71 (10)	C23—C24—H24	120.4 (11)
O1—C5—C4	110.42 (11)	C24—C25—C26	121.04 (14)
C2—C5—C4	105.31 (11)	C24—C25—H25	119.7 (10)
O1—C5—H5	106.3 (9)	C26—C25—H25	119.3 (10)
C2—C5—H5	116.3 (9)	C25—C26—C21	119.67 (13)
C4—C5—H5	111.7 (9)	C25—C26—H26	120.3 (10)
C7—C6—C8	104.76 (11)	C21—C26—H26	120.0 (10)
C7—C6—C1	126.68 (11)	C32—C27—C28	120.90 (12)
C8—C6—C1	128.55 (12)	C32—C27—N2	120.45 (12)
N3—C7—C6	106.86 (11)	C28—C27—N2	118.57 (11)
N3—C7—H7	121.3 (9)	C29—C28—C27	119.68 (12)
C6—C7—H7	131.8 (9)	C29—C28—H28	120.3 (10)
N4—C8—C6	111.26 (11)	C27—C28—H28	120.0 (10)
N4—C8—C15	118.98 (11)	C28—C29—C30	119.83 (13)
C6—C8—C15	129.76 (12)	C28—C29—H29	120.8 (10)
C14—C9—C10	121.14 (13)	C30—C29—H29	119.3 (10)
C14—C9—N3	119.11 (12)	O4—C30—C31	124.41 (13)
C10—C9—N3	119.75 (12)	O4—C30—C29	115.05 (12)
C9—C10—C11	118.96 (13)	C31—C30—C29	120.53 (13)
C9—C10—H10	120.1 (9)	C30—C31—C32	119.40 (12)
C11—C10—H10	120.9 (10)	C30—C31—H31	120.6 (10)
C12—C11—C10	120.31 (14)	C32—C31—H31	120.0 (10)
C12—C11—H11	124.3 (11)	C27—C32—C31	119.65 (12)
C10—C11—H11	115.4 (11)	C27—C32—H32	119.3 (9)
C11—C12—C13	120.17 (14)	C31—C32—H32	121.1 (9)
C11—C12—H12	117.9 (11)	O4—C33—H33A	110.8 (13)
C13—C12—H12	121.9 (11)	O4—C33—H33B	111.9 (12)
C14—C13—C12	119.92 (14)	H33A—C33—H33B	109.9 (18)
C14—C13—H13	118.7 (11)	O4—C33—H33C	105.6 (12)
C12—C13—H13	121.4 (11)	H33A—C33—H33C	107.3 (17)
C9—C14—C13	119.49 (13)	H33B—C33—H33C	111.2 (17)
C9—C14—H14	119.3 (9)		
			
C5—O1—N1—C21	−93.40 (11)	N4—N3—C9—C10	148.20 (12)
C5—O1—N1—C1	36.07 (12)	C7—N3—C9—C10	−31.9 (2)
C7—N3—N4—C8	0.44 (14)	C14—C9—C10—C11	0.2 (2)
C9—N3—N4—C8	−179.62 (11)	N3—C9—C10—C11	179.26 (12)
C21—N1—C1—C6	−151.26 (11)	C9—C10—C11—C12	0.3 (2)
O1—N1—C1—C6	83.28 (11)	C10—C11—C12—C13	−0.5 (2)
C21—N1—C1—C2	88.43 (13)	C11—C12—C13—C14	0.2 (2)
O1—N1—C1—C2	−37.03 (12)	C10—C9—C14—C13	−0.4 (2)
N1—C1—C2—C5	24.76 (12)	N3—C9—C14—C13	−179.52 (12)
C6—C1—C2—C5	−94.18 (12)	C12—C13—C14—C9	0.2 (2)
N1—C1—C2—C3	−88.10 (12)	N4—C8—C15—C20	−145.50 (13)
C6—C1—C2—C3	152.95 (11)	C6—C8—C15—C20	34.8 (2)
C4—N2—C3—O2	−179.52 (12)	N4—C8—C15—C16	32.83 (18)
C27—N2—C3—O2	9.52 (19)	C6—C8—C15—C16	−146.91 (14)
C4—N2—C3—C2	1.03 (14)	C20—C15—C16—C17	0.9 (2)
C27—N2—C3—C2	−169.93 (11)	C8—C15—C16—C17	−177.43 (12)
C5—C2—C3—O2	−178.08 (13)	C15—C16—C17—C18	−1.4 (2)
C1—C2—C3—O2	−66.26 (17)	C16—C17—C18—C19	0.6 (2)
C5—C2—C3—N2	1.36 (13)	C17—C18—C19—C20	0.6 (2)
C1—C2—C3—N2	113.18 (11)	C18—C19—C20—C15	−1.1 (2)
C3—N2—C4—O3	179.04 (13)	C16—C15—C20—C19	0.3 (2)
C27—N2—C4—O3	−9.9 (2)	C8—C15—C20—C19	178.64 (13)
C3—N2—C4—C5	−3.01 (15)	O1—N1—C21—C26	−15.87 (16)
C27—N2—C4—C5	168.09 (11)	C1—N1—C21—C26	−137.45 (12)
N1—O1—C5—C2	−19.58 (12)	O1—N1—C21—C22	170.53 (11)
N1—O1—C5—C4	94.36 (11)	C1—N1—C21—C22	48.95 (16)
C3—C2—C5—O1	114.40 (11)	C26—C21—C22—C23	3.3 (2)
C1—C2—C5—O1	−3.69 (13)	N1—C21—C22—C23	177.07 (13)
C3—C2—C5—C4	−2.97 (13)	C21—C22—C23—C24	−1.2 (2)
C1—C2—C5—C4	−121.07 (11)	C22—C23—C24—C25	−1.2 (2)
O3—C4—C5—O1	66.77 (17)	C23—C24—C25—C26	1.5 (2)
N2—C4—C5—O1	−111.15 (11)	C24—C25—C26—C21	0.6 (2)
O3—C4—C5—C2	−178.40 (13)	C22—C21—C26—C25	−3.0 (2)
N2—C4—C5—C2	3.68 (14)	N1—C21—C26—C25	−176.59 (12)
N1—C1—C6—C7	−33.17 (18)	C4—N2—C27—C32	68.77 (17)
C2—C1—C6—C7	81.74 (16)	C3—N2—C27—C32	−121.14 (14)
N1—C1—C6—C8	145.50 (13)	C4—N2—C27—C28	−108.13 (14)
C2—C1—C6—C8	−99.60 (15)	C3—N2—C27—C28	61.95 (17)
N4—N3—C7—C6	−0.12 (15)	C32—C27—C28—C29	−0.5 (2)
C9—N3—C7—C6	179.94 (12)	N2—C27—C28—C29	176.40 (12)
C8—C6—C7—N3	−0.22 (14)	C27—C28—C29—C30	0.4 (2)
C1—C6—C7—N3	178.70 (12)	C33—O4—C30—C31	−3.9 (2)
N3—N4—C8—C6	−0.58 (14)	C33—O4—C30—C29	176.95 (14)
N3—N4—C8—C15	179.64 (11)	C28—C29—C30—O4	179.37 (12)
C7—C6—C8—N4	0.51 (15)	C28—C29—C30—C31	0.2 (2)
C1—C6—C8—N4	−178.38 (12)	O4—C30—C31—C32	−179.77 (12)
C7—C6—C8—C15	−179.73 (13)	C29—C30—C31—C32	−0.7 (2)
C1—C6—C8—C15	1.4 (2)	C28—C27—C32—C31	0.0 (2)
N4—N3—C9—C14	−32.70 (18)	N2—C27—C32—C31	−176.82 (11)
C7—N3—C9—C14	147.23 (13)	C30—C31—C32—C27	0.6 (2)

### Hydrogen-bond geometry (Å, º)

*Cg*4, *Cg*5, *Cg*6 and *Cg*7 are the centroids of the C9–C14, C15–C20, C21–C26 and C27–C32 benzene rings, respectively.

**Table:**

*D*—H···*A*	*D*—H	H···*A*	*D*···*A*	*D*—H···*A*
C2—H2···*Cg*5^i^	0.992 (16)	2.817 (19)	3.7292 (17)	153.2 (12)
C7—H7···O3^ii^	0.954 (15)	2.329 (15)	3.2666 (16)	167.4 (12)
C22—H22···O4^iii^	0.981 (17)	2.648 (17)	3.4762 (18)	142.3 (13)
C23—H23···*Cg*7^iii^	0.970 (19)	2.97 (2)	3.6086 (18)	124.2 (16)
C28—H28···*Cg*6	0.986 (18)	2.66 (2)	3.4522 (17)	138.1 (14)
C29—H29···O2^iii^	0.950 (18)	2.374 (18)	3.2803 (17)	159.3 (14)
C31—H31···O3^iv^	0.985 (17)	2.360 (17)	3.3133 (17)	162.5 (14)
C32—H32···*Cg*4^v^	0.979 (16)	2.71 (2)	3.3750 (18)	125.3 (12)
C33—H33*A*···N1^v^	0.98 (2)	2.58 (2)	3.393 (2)	140.6 (17)

Symmetry codes: (i) −*x*, −*y*+1, −*z*+1; (ii) −*x*+1, −*y*, −*z*+1; (iii) −*x*+1, −*y*+1, −*z*; (iv) −*x*+1, −*y*, −*z*; (v) *x*, *y*, *z*−1.
